# Feasibility of high‐density electric source imaging in the presurgical workflow: Effect of number of spikes and automated spike detection

**DOI:** 10.1002/epi4.12732

**Published:** 2023-06-01

**Authors:** Ev‐Christin Heide, Daniel van de Velden, David Garnica Agudelo, Manuel Hewitt, Christian Riedel, Niels K. Focke

**Affiliations:** ^1^ Department of Neurology University Medical Center, Georg‐August University Göttingen Germany; ^2^ Institute for Diagnostic and Interventional Neuroradiology University Medical Center, Georg‐August University Göttingen Germany

**Keywords:** amount of IEDs, automation, electric source imaging, focal epilepsy, high‐density EEG, Presurgical evaluation

## Abstract

**Objective:**

Presurgical high‐density electric source imaging (hdESI) of interictal epileptic discharges (IEDs) is only used by few epilepsy centers. One obstacle is the time‐consuming workflow both for recording as well as for visual review. Therefore, we analyzed the effect of (a) an automated IED detection and (b) the number of IEDs on the accuracy of hdESI and time‐effectiveness.

**Methods:**

In 22 patients with pharmacoresistant focal epilepsy receiving epilepsy surgery (Engel 1) we retrospectively detected IEDs both visually and semi‐automatically using the EEG analysis software Persyst in 256‐channel EEGs. The amount of IEDs, the Euclidean distance between hdESI maximum and resection zone, and the operator time were compared. Additionally, we evaluated the intra‐individual effect of IED quantity on the distance between hdESI maximum of all IEDs and hdESI maximum when only a reduced amount of IEDs were included.

**Results:**

There was no significant difference in the number of IEDs between visually versus semi‐automatically marked IEDs (74 ± 56 IEDs/patient vs 116 ± 115 IEDs/patient). The detection method of the IEDs had no significant effect on the mean distances between resection zone and hdESI maximum (visual: 26.07 ± 31.12 mm vs semi‐automated: 33.6 ± 34.75 mm). However, the mean time needed to review the full datasets semi‐automatically was shorter by 275 ± 46 min (305 ± 72 min vs 30 ± 26 min, *P* < 0.001).

The distance between hdESI of the full versus reduced amount of IEDs of the same patient was smaller than 1 cm when at least a mean of 33 IEDs were analyzed. There was a significantly shorter intraindividual distance between resection zone and hdESI maximum when 30 IEDs were analyzed as compared to the analysis of only 10 IEDs (*P* < 0.001).

**Significance:**

Semi‐automatized processing and limiting the amount of IEDs analyzed (~30–40 IEDs per cluster) appear to be time‐saving clinical tools to increase the practicability of hdESI in the presurgical work‐up.

**Key Points**

- We evaluated the effect of number of spikes and automated spike detection on the localization of the high‐density source maximum.
- A semi‐automated spike detection was significantly faster compared to a visual one without affecting source localization results.
- A moderate number of (~30–40) spikes per cluster is advisable to achieve accurate source localization results in our cohort.

## 1 INTRODUCTION

One third of all cases of epilepsy are pharmacoresistant, defined by treatment failure of two tolerated, appropriately chosen and applied anti‐seizure medication (ASM).^1^ In focal epilepsy patients, surgery is an important treatment option with a significantly better outcome in seizure frequency and quality of life compared to continued ASM treatment.^2^, ^3^ The goal of epilepsy surgery is the resection of the epileptogenic zone, to achieve seizure freedom, as well as the sparing of non‐epileptic tissue and tissue with important neuropsychological and neurological function.^4^ Hence, a detailed presurgical evaluation is required.

The presurgical work‐up typically starts with a non‐invasive evaluation (“phase one”), which includes high‐resolution magnetic resonance imaging (MRI), video scalp electroencephalography (EEG), and neuropsychological assessment. Some epilepsy centers also apply high‐density EEG (hdEEG) with electric source imaging (ESI) or, the technically similar, magnetencephalography with magnetic source imaging.^5^ ESI is a back‐projection of the electric activity recorded at the scalp into the (assumed) sources in the brain. This can be done using the measured EEG activity, a forward model and an inverse method such as weighted Minimum Norm Estimation (wMNE).^6^, ^7^, ^8^

High‐density ESI (hdESI) has proven its usefulness in the presurgical evaluation and provides non‐redundant information in a relevant proportion of patients. Compared to low‐density ESI, more accurate source localization was achieved by hdESI.^9^, ^10^ However, in a meta‐analysis including 515 patients by Sharma et al. there was no significant difference in the accuracy of high‐density and low‐density ESI.^11^ These divergent results could be caused by a smooth spatial distribution when a high number of single events in low‐density EEG is used. High‐density EEG may be beneficial for localizing IED averages with a low signal‐to‐noise ratio or IED analysis with a low number of single events. In two interrelated studies by Foged et al. and Duez et al.,^12^ ESI changed the further diagnostic and therapeutic plan in 34% of the patients. It mainly led to an additional intracranial EEG (icEEG) electrode insertion and had greater agreement with icEEG results than visual review by experienced epileptologists alone.^13^ Interestingly, the added icEEG electrodes were the ones to localize the irritative and seizure onset zone (SOZ) in 80% of the cases.^14^ Furthermore, hdESI is a particularly valuable tool for analyzing patients with normal MRI. In a study with 10 operated patients in whom modern MRI sequences failed to provide evidence of an epileptogenic lesion, ESI localized the focus correctly in eight of them.^15^

However, only nine of the 25 surveyed European epilepsy centers reported using ESI in their presurgical evaluation in 2014.^16^ One barrier to a more widespread dissemination of hdESI is its practicability, in particular, the personnel usage for acquisition and review of these datasets. In many centers, only short‐term acquisitions lasting 1–2 hours are done. Long‐term recordings are possible when special electrode systems are used, but these require even more logistical efforts than short‐term recordings (see Section 2.2). Also, physicians should be prepared to invest more time in reviewing long‐term recordings. Despite a huge number of studies on the electrode coverage and the time point of the interictal epileptic discharges (IED) used for hdESI, there is a lack of studies analyzing the amount of IEDs needed and, related to this, how long clinical acquisitions need to be.^9^, ^10^, ^17^ In a recent study by Cox et al.^13^ the authors concluded: “While it is common practice to obtain as many interictal spikes as possible for averaging, there is little data guiding what is an acceptable number of spikes to analyze.” Additionally, Vorderwülbecke et al.^18^ recently published a study on automated interictal source localization based on hdEEG, which provided meaningful information in the majority of cases. However, spatial downsampling to low‐density ESI did not decrease the accuracy. As the IED detection in big data files such as hdEEG is a very time‐consuming process, a semi‐automated IED detection could contribute to an improved practicability.

In this study, we have systematically evaluated the effect of semi‐automated IED detection by the EEG analysis software Persyst on the accuracy of hdESI and the operator time needed. Moreover, we analyzed the influence of IED quantity on the localization of hdESI. This could help to increase the efficiency of hdESI in the presurgical work‐flow and guide centers when establishing or optimizing their hdEEG programs.

## 2 METHODS

### 2.1 Clinical data acquisition

The patients with completed presurgical non‐invasive evaluation between September 1, 2017 and February 1, 2021 were selected retrospectively from the “epilepsy surgery database” of the department of Neurology, University Hospital of Göttingen. Inclusion criteria were defined as follows: (1) diagnosis of pharmacoresistant focal epilepsy, (2) presurgical evaluation with 256‐channel hdEEG (≥105 min) recording and high‐resolution 3 T MRI *with* T1 or MPRAGE sequences, (3) ≥10 IEDs in hdEEG, (4) subsequent first epilepsy surgery with good outcome (Engel 1),^19^, ^20^ and (5) postsurgical MRI with T1 sequences. We established the following exclusion criteria: (1) multifocal epilepsy and (2) prior epilepsy surgery. This retrospective study was approved by the local ethics committee at the University Hospital Göttingen (number 3/7/22).

### 2.2 Acquisition of hdEEG

HdEEG recordings were performed on the video‐EEG monitoring unit using a GES400 system (Electrical Geodesics, Inc., now Magstim EGI, Eugene, USA, at 250 or 1000 Hz sampling rate). In patients with less than 10 IEDs per hour in the preceding low‐density EEG a long‐term hdEEG (≥03:00 h) was performed, whereas in patients with high more than 10 IEDs per hour in the preceding low‐density EEG a short‐term hdEEG (≥01:45–03:00 h) was deemed clinically sufficient. The short‐term hdEEG was recorded with the HydroCel Geodesic Sensor Net 130 (Electrical Geodesics), where interconnected sponge electrodes are soaked in a saline solution and placed on the patient's head without a paste. In contrast, long‐term hdEEG requires a longer application time, as a scalp preparation and placement of a conductive paste under each of the 256 electrodes of the HydroCel Geodesic Sensor Net (130 LT) is needed. Electrode‐skin impedances were kept at <15 kΩ.

### 2.3 Visual versus semi‐automated IED detection in hdEEG

The full‐length, filtered hdEEG was displayed by EGI Net Station Review. First, the IEDs were detected purely visually from the beginning to the end by an EEG‐experienced clinician (E.‐C. Heide, aware of clinical information). All IEDs were marked on the peak using a conventional display (longitudinal bipolar or average montages of the 10–20 electrode array plus extended electrodes in the temporal chain, corresponding to IFCN recommendations^21^) and visually classified into different groups based on their topography, at lobar resolution. The time needed for visual review of the hdEEG was measured and rounded up in 15‐minute steps.

Additionally, the full‐length, filtered hdEEG was processed by Persyst Spike Detector P14 (Persyst; non‐clinical use, version 14, Rev. C). We excluded IEDs with a spike probability measure of less than 0.9, corresponding to the “low sensitivity” settings of the software.^22^ The remaining IEDs were grouped automatically into different clusters according to the electrode with the highest amplitude. Each cluster was then sorted by the IED amount and displayed as 2‐second event epochs. Semi‐automated IED detection includes the review of these epochs by the same clinician (E.‐C. Heide) at least 6 months after visual scoring using an extended average reference montage as mentioned above. Automatically detected events that did not meet IED criteria such as artifacts and physiological EEG patterns were excluded. The operator time needed for reviewing all 2‐second event epochs was measured and rounded up in 15‐minute steps.

In order to compare the visual and semi‐automated IED detection, sensitivity, positive predictive value, success rate and Cohen's κ (kappa) were determined. Sensitivity was defined by the amount of visual IEDs detected also by Persyst (sensitivity [%] = (*n* (IEDs detected visually AND by Persyst)/*n* (IEDs detected visually)) × 100%), whereas positive predictive value was the amount of IEDs among all detected events. Success rate was described as the probability to detect at least 30 IEDs among all events of one cluster (e.g. success rate of 1st cluster [%] = (*n* (patients with at least 30 IEDs in the 1st cluster)/*n* (all patients)) × 100%). In addition, the Cohen's κ for inter‐rater agreement analysis was used to observe the agreement reliability between semi‐automated or fully‐automated IED detection and purely visual detection. It was calculated as

$$\kappa =\frac{\mathrm{Pr}\left(a\right)-\mathrm{Pr}\left(e\right)}{1-\mathrm{Pr}\left(e\right)}$$

where Pr(a) is the relative observed agreement between selection conditions, and Pr(e) is the hypothetical probability of chance agreement.^23^, ^24^, ^25^

### 2.4 Determination of surgical reference

Postsurgical T1 was co‐registered to the presurgical T1 and the resection zone was marked automatically. Details are provided as supplementary information. The extent of the resection was determined clinically including non‐invasive and, for some patients, invasive EEG diagnostics and surgical access routes. Given the fact that only patients with Engel 1 postsurgical outcome were included, it can be assumed that the resection zone contains the epileptogenic zone or, at least, clinically relevant propagation areas.

### 2.5 Source reconstruction

HdESI was performed with only one cluster of visually as well as semi‐automatically detected IEDs per patient. The cluster with the highest number of visually detected IEDs and a comparable cluster of semi‐automatically detected IEDs was chosen. For this reason, the semi‐automated IEDs clustered automatically by Persyst were grouped visually by hemisphere and lobar resolution to allow comparison with visual hdESI.

**FIGURE 1:**
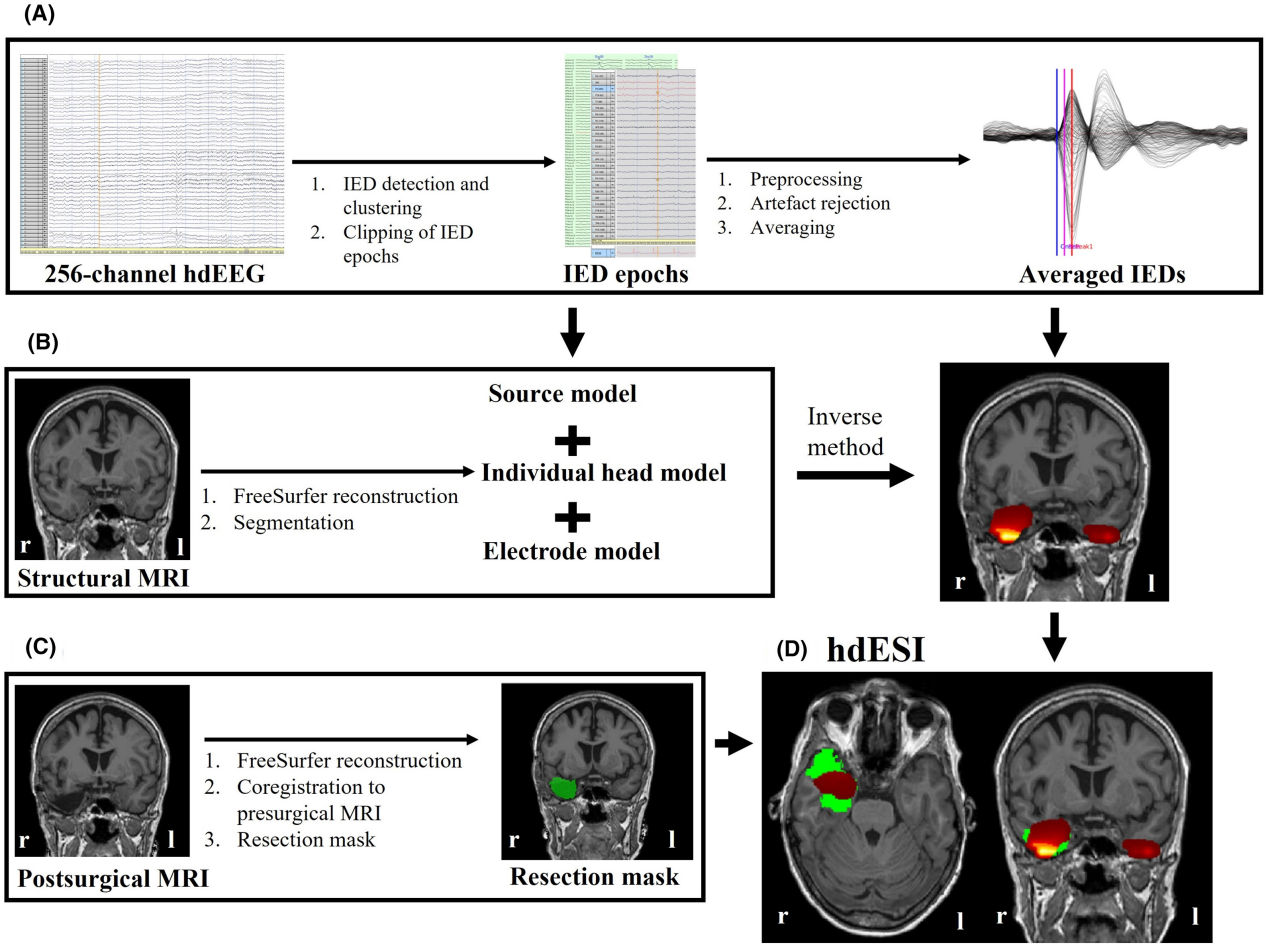
Flow‐chart of the hdEEG processing steps. Review of the EEG recordings included a visual versus semi‐automated marking of IEDs. The 2‐s IED epochs were then preprocessed, cleaned of artifacts and averaged within each cluster to improve signal‐to‐noise ratio (A). Based on the individual structural MRI and the hdEEG a head model, source model and electrode model were created for each patient (B). Furthermore, a resection mask (green) was performed based on the postsurgical MRI (C). HdESI maximum (yellow area within read area) was compared to the resection mask (D).

Epochs containing the visually or semi‐automatically detected IEDs (±2 s) were clipped and exported. They were further processed and analyzed with Fieldtrip (https://www.fieldtriptoolbox.org/, version fieldtrip‐20 191 127) running in Matlab (version 9.0, R2018b, Mathworks Inc.). wMNE as well as the time point at 50% rising phase were chosen for the source reconstruction according to literature.^7^, ^8^, ^26^, ^27^ In addition to the measured EEG activity and the inverse method, a forward model is needed. The forward model is based on the head model, the source model and the electrode model. To create the individual head model, the FreeSurfer processed, intensity normalized MRI of each subject was used. A regular 5 mm volumetric grid was constructed in the CAT12 template (CAT12; Christian Gaser 2018, http://www.neuro.uni‐jena.de/cat/) in the MNI space. Those standardized volumetric grid points were transformed back to the individual anatomical space by using the inverted DARTEL transformation (DARTEL; SPM12; https://www.fil.ion.ucl.ac.uk/spm/software/spm12/). These volumetric grid points were further used for the source space for each subjects' forward model. An individual boundary element model with three layers of different conductivities (scalp: 0.33S/m, skull: 0.004S/m, and brain: 0.33S/m) was constructed using the “dipoli” method implemented in Fieldtrip. For the sensor model, the sensors were spatially aligned to the anatomical T1 using anatomical landmarks of fiducial positions (left/right preauricular point, nasion). Details on hdESI processing steps are provided as supplementary information. An overview of the processing steps is displayed in Figure 1(A–D).

Source maxima were compared to the sublobe(s) and to the resection itself. The gray matter was divided visually into 19 “sublobes” per hemisphere based on the Lausanne parcellation atlas.^18^ In order to evaluate the effect of IED amounts on hdESI accuracy, the shortest Euclidean distance between the resection zone and the maximum amplitude of hdESI was measured for different amounts of IEDs of each patient. Furthermore, the Euclidean distance between the intraindividual hdESI maximum when all IEDs of an individual were included and the hdESI maximum of reduced amounts of IEDs was determined.

### 2.6 Statistical analysis

Statistical analysis was performed using GraphPad Prism (version 6, GraphPad Software) and Matlab. Differences were analyzed with Student's *t*‐test (or Mann–Whitney U‐Test) and shown as means ± standard deviations (SD) (or medians ± interquartile ranges) when data were normally (not normally) distributed. Correlations of nonparametric data were analyzed using the Spearman's rank correlation coefficient (*r*_*s*_). Cohen's *κ* was calculated using Matlab, and its interpretation followed the recommended nomenclature.^23^, ^24^

*P*‐values <0.05 were considered statistically significant. They were corrected for multiple comparisons according to Holm–Bonferroni method.

## 3 RESULTS

### 3.1 Patient cohort and IED frequency

A cohort of 22 patients out of 67 patients was identified retrospectively from the “epilepsy surgery database” between September 2017 and February 2021. All of the identified patients met the inclusion criteria (Figure S1). None of them needed to be excluded. The hdEEG acquisition length was 146 ± 36 min in short‐term hdEEG and 914 ± 135 min in long‐term hdEEG. Five out of 22 patients were diagnosed with extratemporal lobe epilepsy due to low‐grade, developmental, epilepsy‐associated brain tumors (LEAT), focal cortical dysplasias (FCD) or cavernomas, whereas the remaining 17 patients had temporal lobe epilepsy, mostly with hippocampal sclerosis as the underlying cause. An overview of clinical data and main source imaging results is displayed in Tables 1 and S1.

**TABLE 1:** Patient demographics, clinical characteristics and main source imaging results.

		All patients
Group size		22
Sex	Female	8 (36%)
	Male	14 (64%)
Duration epilepsy to surgery (y)		15,45 [14,42]
Age at surgery (y)		40,41 [12.38]
Seizure frequency one y before surgery (seizures per mos)		8.92 [10.83]
Structural MRI	Lesional	15 (68%)
	Non‐lesional	7 (32%)
HdEGG length (hh:mm:ss)	Short‐term	02:25:34 [00:36:07]
	Long‐term	15:14:01 [02:15:13]
HdEEG recording	Short‐term	4 (18%)
	Long‐term	18 (82%)
SOZ location (TLE; ETLE)	TLE	17 (77%)
	ETLE	5 (23%)
SOZ hemisphere	Left	13 (59%)
	Right	9 (41%)
Surgery	AH + ATR	12 (54.5%)
	AH	2 (9%)
	LiTT	1 (4.5%)
	Other	7 (32%)
Histology	HS	7 (31.8%)
	GG	3 (13.6%)
	FCD	2 (9.1%)
	Cavernoma	3 (13.6%)
	Normal cortex/Gliosis	6 (27.3%)
Surgical outcome at last follow‐up (≥6 mos; Engel classification)	IA	16 (72.73%)
	IB	1 (4.54%)
	ID	5 (22.73%)
Post‐op follow‐up (mos)		18.27 [9.08]
Spike counts (IEDs/patient)	Visual	74 [56]
	Semi‐automated	116 [115]
Mean Euclidean distance (mm)	Visual	26.07 [31.12]
	Semi‐automated	33.64 [34.75]
Sublobar concordance	Visual	13 (61.9)
	Semi‐automated	12 (57.14)
Lobar concordance	Visual	14 (66.7)
	Semi‐automated	13 (61.9)

*Note*: All patients had refractory focal epilepsy, all of them with Engel 1 outcome after epilepsy surgery. Data are given as *n* (%) or as mean [standard deviation].

Abbreviations: AH, amygdalohippocampectomy; ATR, anterior temporal lobe resection; ETLE, extratemporal lobe epilepsy; FCD, focal cortical dysplasia; GG, ganglioglioma; hdEEG, high‐density EEG; HS, hippocampal sclerosis; LiTT, laser interstitial thermal therapy; mos, months; SOZ, seizure onset zone; TLE, temporal lobe epilepsy; y, year(s).

The detected IED frequency correlated with the seizure frequency before epilepsy surgery (visual detection: *r*_*s*_ = 0.47, *P* = 0.03; semi‐automated detection: *r*_*s*_ = 0.59, *P* = 0.008; *n* = 22), but not with epilepsy duration. Cavernomas and LEATs showed a tendency to a lower IED frequency compared to hippocampal sclerosis regarding visual, but not semi‐automatically detected IEDs (2.93 (0.96–4.37 IED/h) versus 5.31 (4.36–22.54 IED/h) (median [IQR]), *P* = 0.096, Mann–Whitney U‐Test, *n* = 12). There was no significant difference in IED frequency between extratemporal and temporal lobe epilepsies and no significant correlation between IED frequency and ASM reduction from admission to time of hdEEG.

### 3.2 Comparison of visual and semi‐automated IED detection

There was no significant difference in the number of IEDs between visually versus semi‐automatically marked IEDs (visual detection: 74 ± 56 IEDs/patient versus semi‐automated detection: 116 ± 115 IEDs/patient, *P* = 0.13, *n* = 22). However, the difference might become significant with an increasing sample size. The visual IEDs were divided in either one, two or three group(s) (*n* = 11, 7, 4, respectively), according to their lobar topography. The semi‐automatically detected IEDs were classified according to the electrode with the highest amplitude into a mean of eight clusters. Overall, Persyst had a mean IED sensitivity of 77.12 ± 21.82%, defined by the ratio of visually detected IEDs also found by Persyst. The mean positive predictive value of Persyst was low with 4.11 ± 2.65%. The other detected events consisted of technical and biological artifacts and physiological EEG patterns such as wicket spikes and sleep patterns. The Cohen's *κ* for visual versus purely automated IED detection is 0.08, indicating a slight level of agreement. In contrast, the Cohen's *κ* for visual versus semi‐automated IED selection is 0.65, corresponding to a substantial level of agreement.

**FIGURE 2:**
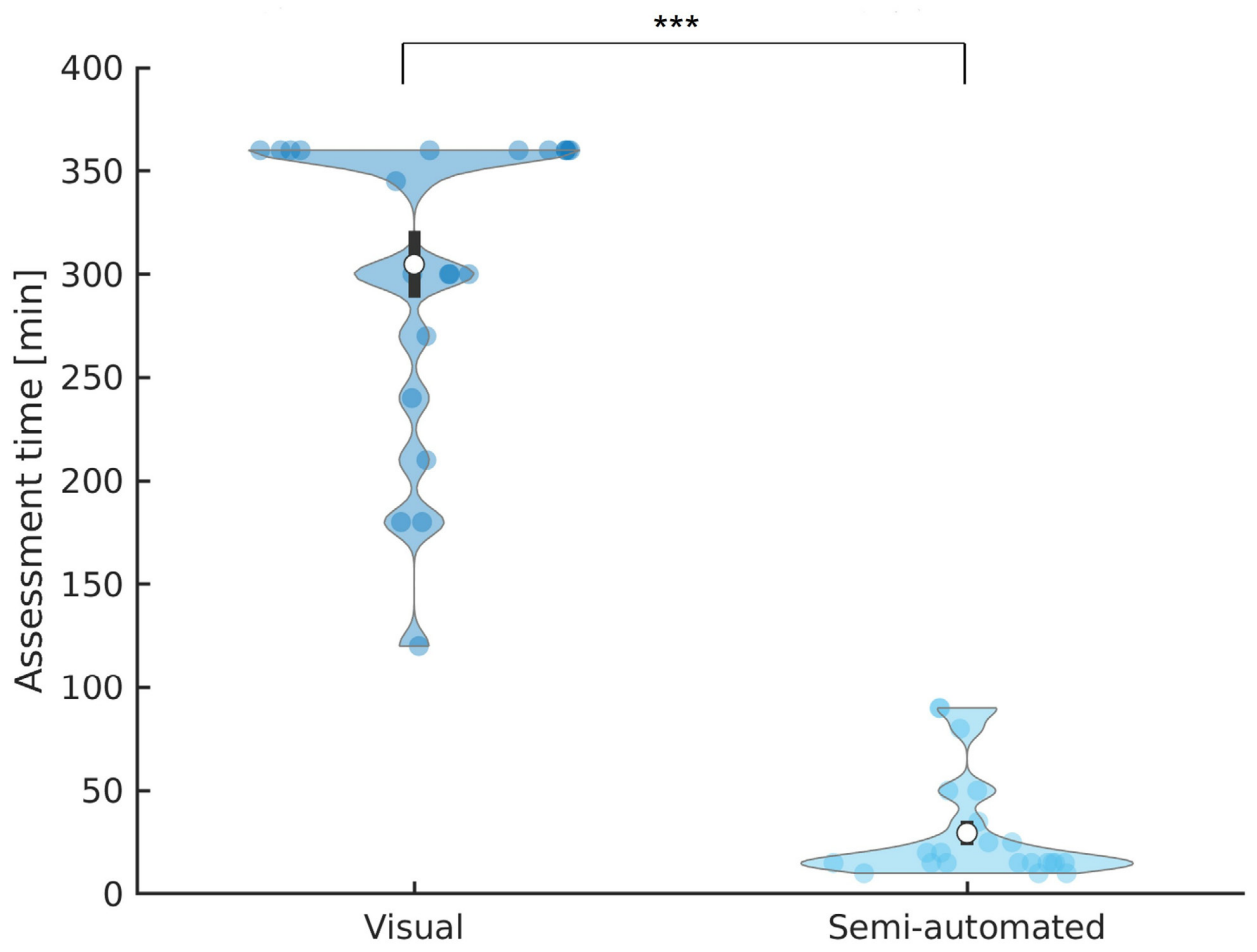
Time efficiency of visual IED marking compared to semi‐automated IED detection with Persyst. The mean assessment time to score the full length hdEEG was shorter with semi‐automated Persyst detection compared to purely visual marking (305 ± 72 min vs 30 ± 26 min, *n* = 22, *P* < 0.001, *t*‐test). Data is shown in mean (white circle) ± standard deviation (black lines) and data density (blue area). Statistically significant group differences are marked with an asterisk (***P < 0.001).

When analyzing only Persyst detected clusters grouped by the electrode with the highest amplitude, the 10 most prevalent clusters included a mean number of 1951 ± 219 single events and a mean IED sensitivity of 86.64 ± 15.77%, defined by the amount of semi‐automated detected IEDs among all Persyst detected events (Figure 2A,B). There was a significantly higher IED sensitivity of the first four clusters compared to the one of the fifth to tenth cluster (62.75 ± 35.66% versus 23.88 ± 24.12%, *P* < 0.001, *t*‐test). The success rate to detect at least one event cluster with more than 30 IEDs increased from 45.45%, when considering the most prevalent cluster only, to 86.36%, when considering the first 10 clusters (Figure 2C). The mean success rate of a 30 IED‐cluster detection was significantly higher in the first four clusters compared to the fifth to tenth cluster (50% vs 4.55%, *P* < 0.001, *t*‐test).

The time to select all IEDs in the full‐length hdEEG was significantly shorter with the semi‐automated compared to the visual marking by 275 ± 46 min (305 ± 72 min vs 30 ± 26 min, *P* < 0.001, *t*‐test, *n* = 22; Figure 2).

### 3.3 Effect of semi‐automated IED detection on hdESI

**FIGURE 3:**
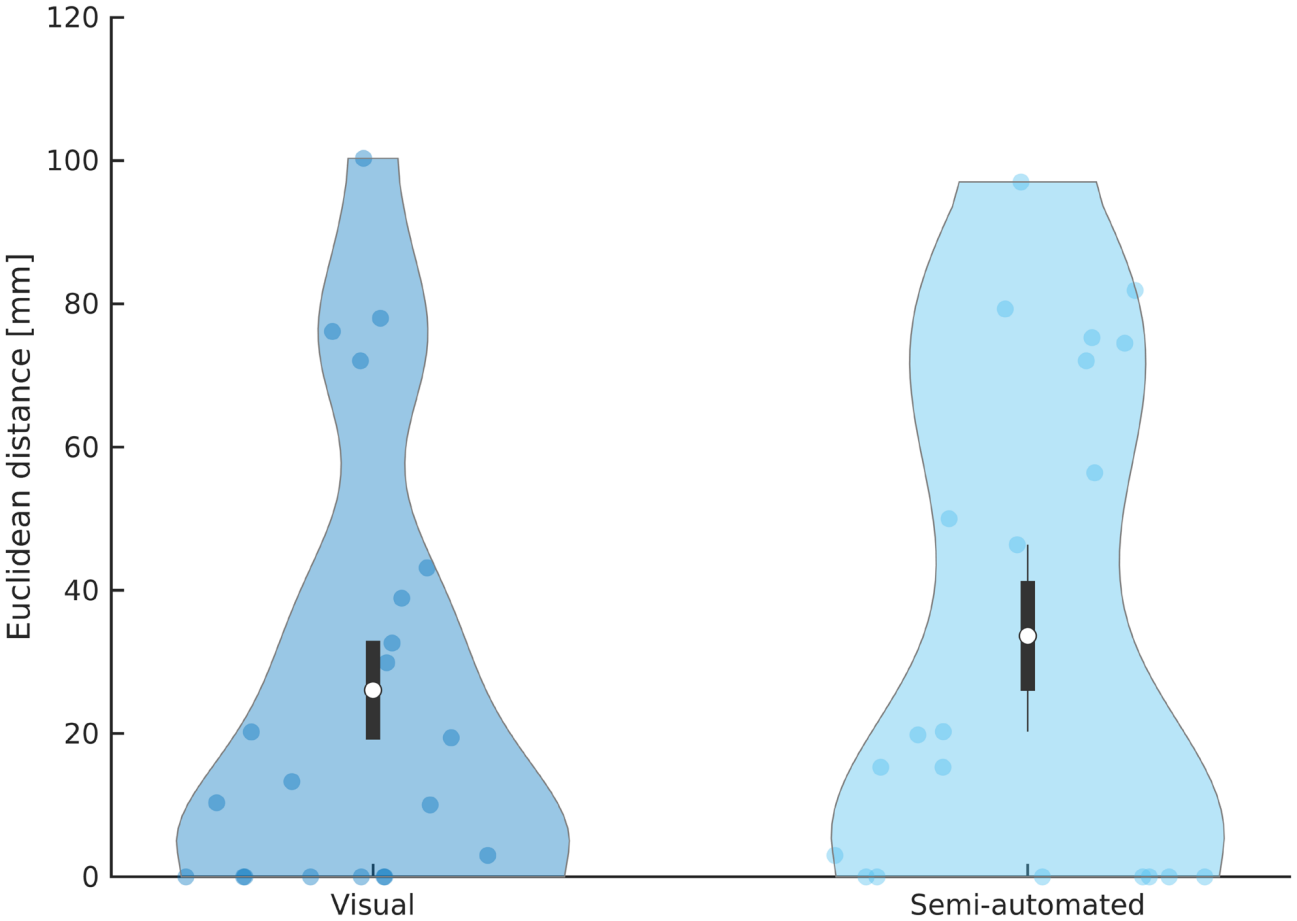
Effect of visual versus semi‐automated IEDs on hdESI accuracy. There was no significant difference between the mean Euclidean distance of the visual versus semi‐automated Persyst detection compared to purely visual marking (*P* = 0.87). Data is shown in mean (white circle) ± standard deviation (black lines) and data density (blue area).

**FIGURE 4:**
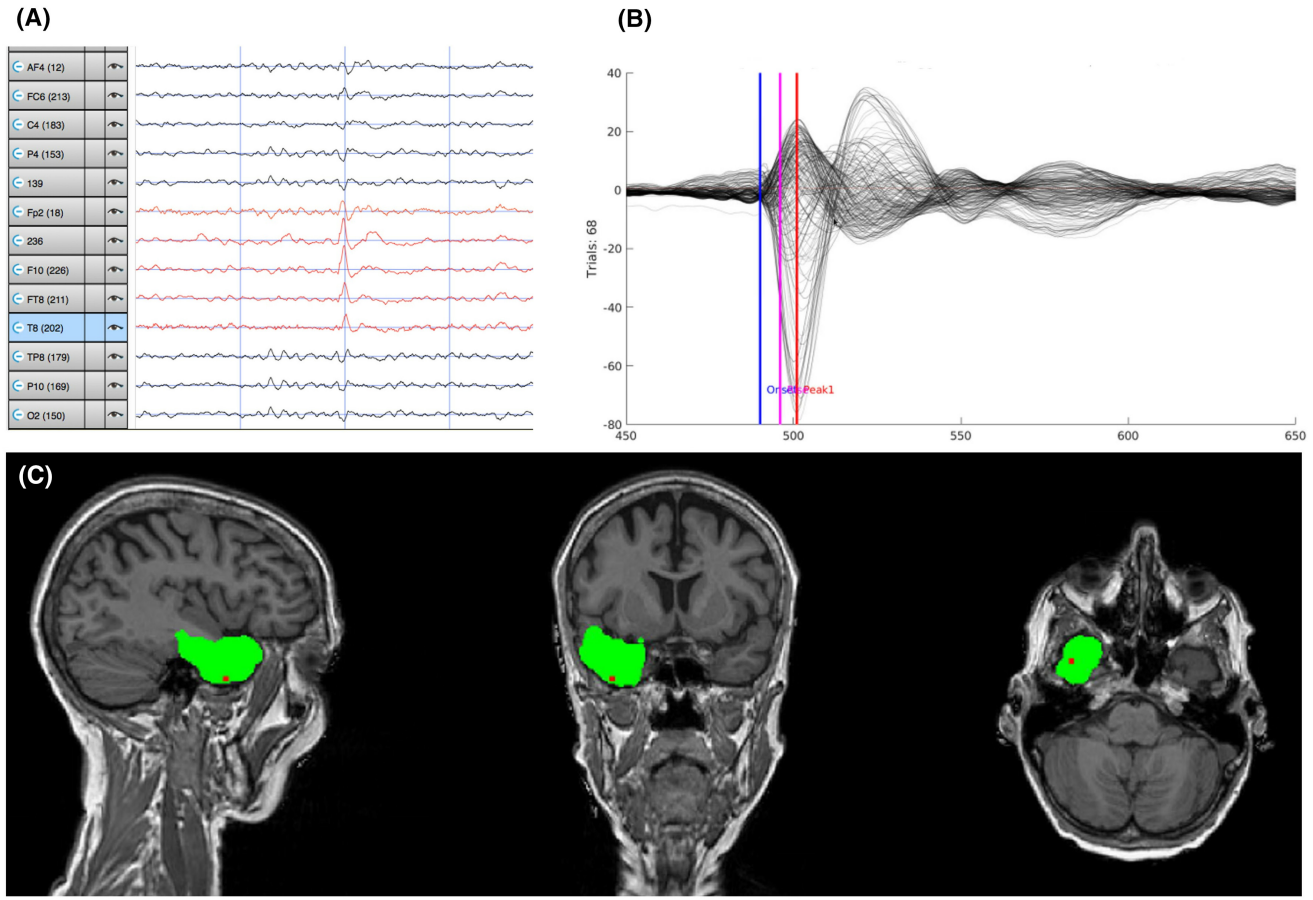
ESI results of an MRI positive patient with 256‐channel hdEEG. The patient was Engel class 1A at 1‐year follow‐up after anterior temporal lobectomy. Pathology confirmed hippocampal sclerosis. Single IED on the right temporal electrodes displayed on an extended average montage (A). An average of 68 IEDs (B). HdESI with resected volume (green) and the maximum amplitude of ESI (red point) (C). wMNE sublobar localization was right temporal. Euclidean distance was 0 mm with source maximum being in the resection zone.

The mean distance between the resection zone and the hdESI maximum was 26.07 ± 31.12 mm for visual detection and 33.64 ± 34.75 mm for semi‐automated detection, without significant difference (*P* = 0.46, Figure 3). Similarly, the sublobar concordance was around 60% in both groups (Table 1). A subgroup analysis of distances and concordance rates is given in Table S2. In patients with temporal SOZ (*n* = 16) the distance was significantly lower than in patients with extratemporal SOZ (*n* = 5) (visual: 16.07 ± 21.88 mm vs 58.06 ± 37.00 mm and semi‐automated: 22.81 ± 28.74 mm vs 68.30 ± 31.15 mm; *P* < 0.014, *t*‐test). There was a significantly higher distance for cavernomas and LEATs compared to hippocampal sclerosis (visual: 47.65 ± 26.00 mm vs 2.22 ± 4.16 mm and semi‐automated: 54.15 ± 41.94 mm vs 10.78 ± 18.42 mm [*n* = 11]; *P* ≤ 0.047, *t*‐test). Cavernomas and LEATs had an extratemporal SOZ in three out of five patients. There was no significant difference in the distance between the MRI positive and negative group. An example of hdESI results of an MRI positive patient is shown in Figure 4.

### 3.4 Effect of IED amount on hdESI

**FIGURE 5:**
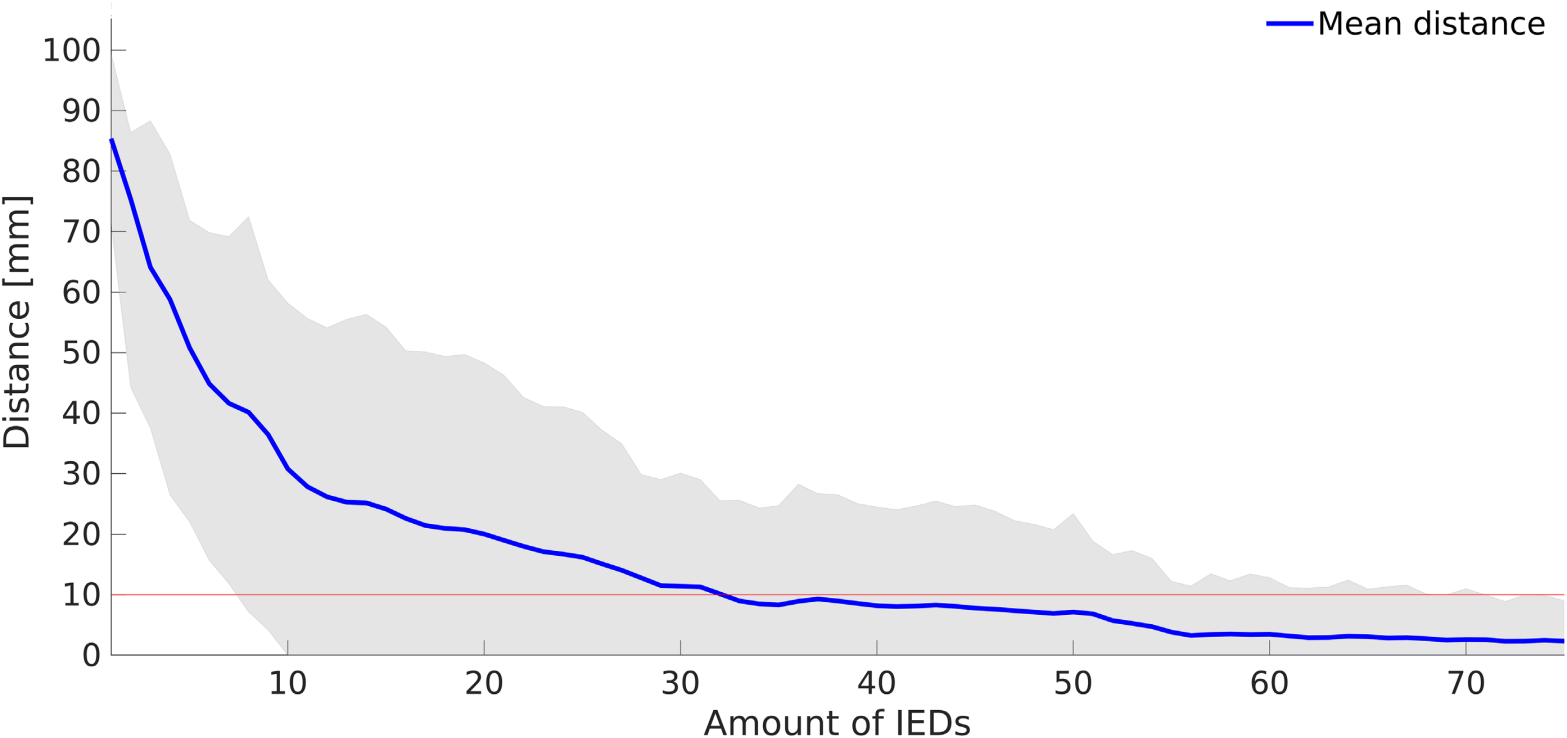
Effect of IED amount on localization of hdESI maximum. The mean Euclidean distance between the source reconstruction of the full individual IED amount and the source reconstruction of reduced IED amounts of the same patient was lower than 1 cm above a mean number of 33 IEDs (red line: Euclidean distance of 1 cm; blue line: overall mean distance; gray area: standard deviation).

The mean distance between hdESI maximum of the full IED amount and the one of reduced IED amounts of the same patient was lower than 1 cm when a mean number of at least 33 IEDs were included (Figure 5). Similarly, the distance between resection zone and hdESI maximum did not change more than 1 cm above a mean number of 20 ± 26 IEDs.

For intraindividual comparison, all patients with a maximum of 30 or more IEDs (*n* = 15) were selected and mean distance between hdESI maximum of the full amount of IEDs and hdESI maximum of 30 and 10 randomly chosen IEDs of the same individual were compared. In this subgroup analysis, we found a significantly shorter distance when 30 IEDs are included in comparison to 10 IEDs (4.21 ± 18.36 mm vs 35.98 ± 32.55 mm, *P* < 0.001, *t*‐test). Accordingly, we found a significantly shorter distance to the resection zone when 30 IEDs are included compared to 10 IEDs (6.97 ± 31.40 mm vs 31.54 ± 28.85 mm, *P* = 0.03, *t*‐test).

## 4 DISCUSSION

In this study we addressed two main issues of practicability of interictal hdESI, namely semi‐automated IED detection and minimum amount of IEDs needed. We found that semi‐automated IED detection can reduce review time without affecting the localization accuracy of hdESI and that 30–40 IEDs are advisable per cluster.

### 4.1 Effect of semi‐automated IED detection on hdESI

Most hdESI applications focus on interictal events, hence, the detection of IEDs is an essential part of clinical diagnostics. IED selection can be made purely visually or semi‐automatically by different software. Here, Persyst Spike Detector P14 C was able to identify 77.12 ± 21.82% of the visually detected IEDs. Moreover, sublobar concordance and mean distance between resection zone and hdESI of the visually and semi‐automatically detected IEDs was without significant difference.

Concerning the sensitivity of IED detection, a study by Reus et al.^28^ compared three commercial automated spike detection software packages (Persyst, Encevis, BESA). IED detection by Persyst was better than the other two software packages concerning sensitivity and similarity to human review. Accordingly, it has been shown, that Persyst was statistically noninferior to the skilled human readers in standard 10–20 system electrode recording sites plus sub‐temporal electrodes.^22^ The average level of pairwise spike marking sensitivities of three skilled human EEG readers was about 45%, and comparable to a sensitivity of 43.9% of Persyst. Very recently, an artificial intelligence‐based computer algorithm had a sensitivity of 89% for identifying EEGs with IEDs recorded from patients with epilepsy, but human expert supervision was still necessary for confirming the clusters of detected IEDs.^29^ In previous studies, the reported average pairwise reader spike sensitivities were comparably high with 52% and 70%, respectively, mostly due to utilizing brief records containing high spike density and largely demonstrative spike types.^30^, ^31^

There are only a few studies addressing the application of automated IED detection in hdEEG, which allows more accurate ESI results than low‐density ESI.^9^, ^10^, ^32^ Vorderwülbecke et al.^18^ recently published a study on the validity of automated hdEEG IED detection by Persyst and hdESI. In contrast to our study, these authors did not compare automated with visual IED detection. Instead, automatically detected clusters were reviewed if they were IEDs or artifacts. A semi‐automated spike detection sensitivity of 73% and spike detection rate of 38% was described. Compared to our results, Heers et al. reported a good concordance of the SOZ with the most frequently and visually detected IED types in hdEEG, but not with (semi‐) automatically detected IEDs in hdEEG. One reason for the difference could be the newer version of Persyst we used compared to the one used by Heers et al.^33^

The time to score the whole hdEGG was significantly shorter with Persyst. Given the increased number of IEDs available in long‐term recordings,^34^ this can balance the higher workload needed for reviewing theses datasets. To our knowledge, this is the first study comparing the time between the two different detection methods.

Regarding hdESI results of visually and automatically detected IEDs, the mean sublobar concordance rate of temporal lobe epilepsies was in line with current studies (visual: 75.00% and semi‐automated: 68.75%, *n* = 16). However, the concordance rate for extratemporal lobe epilepsies was lower (visual and semi‐automated: 20%, *n* = 5). In semi‐automated hdESI, a sublobar concordance rate of 75% in a 256‐channel setting on half‐rise of the averaged IEDs of patients with a favorable postsurgical seizure outcome (ILAE 1 and 2, see Vorderwülbecke et al., table S3) was reported.^18^ Visual hdESI studies such as Toscano et al. 2020 and Lascano et al. found a similar sublobar concordance rate of patients with a favorable postsurgical seizure outcome (Engel 1) of 75% and 88%, respectively.^35^, ^36^ Interestingly, the sensitivity was lower when fully automatically interictal ESI was performed. For example, Iachim et al.^37^ described a sensitivity of 53% based on sublobar concordance between low‐density ESI localization and irritative zone in SEEG,^37^ whereas van Mierlo et al.^38^ reported a sensitivity of 70% based on concordance between low‐density ESI localization and resection zone. A comparison of both methods showed a sensitivity of 88% in the semi‐automated approach versus 60% in the fully automated method.^39^ The mean Euclidean distance between source maximum and resection zone is rarely mentioned in the literature and there is no data on the distance in automated hdESI. Vorderwülbecke et al. 2020 and Carboni et al. 2022 (same cohort) subdivided the Euclidean distance between the source maximum and the resection zone for patients with favorable postsurgical outcome (Engel 1 and 2, *n* = 30) into three groups (<10 mm, <20 mm, >20 mm).^26^, ^40^ The proportion of each group in the cohort was given for temporal and extratemporal lobe epilepsies (temporal: 81%, 16%, 5% and extratemporal: 45%, 10%, 45%, see Vorderwülbecke et al. 2020, Figure 3 and Data S2). Our results showed slightly higher distances for temporal lobe epilepsies when subdividing them into the same three groups (visual: 56%, 19%, 25% and semi‐automated 50%, 19%, 31%) and much higher distances for extratemporal lobe epilepsies (visual: 20%, 0%, 80% and semi‐automated: 0%, 20%, 80%). As the amount of IEDs was lower than in our cohort (median: 29 IEDs/patient and mean: 45 IEDs/patient in Vorderwülbecke et al. 2020, see Data S2, *n* = 21 with 9 of unknown IED amount), this difference is probably not explained by the amount of IEDs. Possible reasons for the higher distances in our cohort, especially in extratemporal lobe epilepsies, include the different inverse method or the different sample composition.^40^ Additionally, the extratemporal subgroup was very small. However, in terms of the main question of our work, there was no significant difference in the distance between semi‐automated and visual hdESI of extratemporal lobe epilepsies as well as of temporal lobe epilepsies in our cohort. When comparing interictal ESI with the resection zone, the limitations of interictal ESI should be considered. Interictal ESI mainly represents the irritative zone, which may or may not overlap with the epileptogenic zone and SOZ.^9^, ^14^

### 4.2 Effect of IED amount on hdESI

Performing hdESI requires expertise and time, especially when a long recording (several hours to several days) is needed to get enough IEDs for ESI. While it is common practice to obtain as many IEDs as possible for averaging, there is little data guiding what is an acceptable number of IEDs to analyze. In our cohort, the difference between 30 and 10 IEDs was more than 30 mm when referenced to hdESI maximum with the full amount of IEDs. However, the difference between 30 IEDs and 10 IEDs was <5 mm when referenced to the resection zone. Depending on the different need of accuracy this could help the clinician to determine the IED amount.

To our knowledge, this has been the first study to analyze this question in hdEEG (256 channels), although many studies expressed the clinical need^13^, ^17^ In previous studies, the exact number of IEDs used for hdESI differed widely. Foged et al.,^12^ Sohrabpour et al.^10^ and Toscano et al.^35^ used a minimum of five, 15 or 10 IEDs, respectively, whereas Plummer et al.^41^ and Heers et al.^42^ performed hdESI on an average number of 48 and 107 IEDs, respectively. In former low‐density EEG studies, the averaging of identical spikes improved source localization accuracy compared to ESI performed on single spikes.^43^, ^44^ Considering our results, a number of approximately 30–40 IEDs is sufficient to get accurate hdESI results. However, it should be mentioned, that an IED number higher than 30–40 would decrease localization errors and affect accuracy. The question of similarity concerning localization and morphology of IEDs clustered in one group still remains unclear and needs to be studied in detail.

### 4.3 General aspects of hdESI in our cohort

Interestingly, the localization of the SOZ has an impact on hdESI. In our cohort, patients with temporal SOZ had significantly shorter distances between ESI maximum and resection zone than patients with extratemporal SOZ (visual: 16.07 ± 21.88 mm vs 58.06 ± 37.00 mm; semi‐automated: 22.81 ± 28.74 mm vs 68.30 ± 31.15 mm). The effect size of the localization of the SOZ on the distance to the resection boundary was higher than the effect of an increasing number of IEDs above a number of 30–40 IEDs. In line with our results, Cox et al.^13^ previously found a higher rate of complete concordance of ESI with icEEG on a sublobar level in temporal compared to extratemporal lobe epilepsy.^9^, ^13^ The reason for this is not clear, but might be a consequence of different IED topography, different number of IEDs analyzed, the forward model, the inverse method or better scalp EEG accessibility of temporal areas due to thinner bone structures. Another reason could be, that the inclusion of very caudal electrodes as in the 256‐channel set‐up lead to a shift of the hdESI maximum towards the temporal lobes even in extratemporal epilepsies.^26^

There is little data guiding on how similar spikes should be morphologically in order to be grouped. An additional biasing factor could be etiology of the epilepsy. As extratemporal lobe epilepsies were either LEATs, FCDs or cavernomas, this could have influenced the size of resection and the amount and morphology of IEDs.^45^, ^46^ However, the group size in our cohort was too small to study this in detail. Patients with non‐lesional and lesional MRI had 86% (6/7) and 57% (8/14) sublobar concordance rates, respectively. This is particular interesting, keeping in mind that hdESI is a very important tool for MRI‐negative epilepsies and should be studied in a larger patient sample. In line with our results, a study of Brodbeck et al. reported the correct localization of the epileptogenic focus by ESI in 80% of patients with normal MRI.^15^ In a larger study cohort (*n* = 74), the concordance rate of ESI with intracranial EEG was higher in MRI‐negative epilepsies compared to MRI‐positive epilepsies.^47^

### 4.4 Limitations

Our study has limitations given the retrospective nature and logistical constraints. The clinician scoring the hdEEGs is part of the epilepsy team and was not blinded for clinical information. Furthermore, the same hdEEG was scored twice by the same reader, purely visually and semi‐automatically, which could have influenced scoring time. By having an interval of at least 6 months in between the two scorings, we argue that the impact of this factor is not strong. An additional limitation is the use of only one inverse method and IED timepoint. However, we chose a common inverse method and timepoint. A previous study by Pellegrino et al. 2020 reported lowest distance between the map maximum and epilepsy focus in MNE.^7^ Other studies, found other preferable source reconstruction methods, although differences were often slight.^40^ Moreover, although the IED scoring and experimental ESI analysis was not part of the clinical evaluation, all patients received clinically driven ESI (done by other clinicians and using other software), which may have had an influence on indicating surgery especially in non‐lesional patients. However, ESI is just one of many modalities used in the (pre‐)surgical decision making in our program. As the Matlab toolbox we used may not be suited for all clinical workflows, further studies that compare our results to common clinical software packages are relevant. Prospective and multi‐center studies on details of clinical hdEEG use such as the choice of the inverse method or the software package and the effect of IED clusters are needed.

## 5 CONCLUSIONS

This study provides evidence for better practicability of hdESI. In particular, we could show that 30–40 IEDs per cluster are advisable. Moreover, semi‐automated IED selection dramatically improved the scoring time needed without significantly affecting hdESI results.

Overall, hdESI is a valuable contributory technique in surgical planning. Our results can help to improve the efficiency of hdESI and increase its use in epilepsy centers.

## AUTHOR CONTRIBUTIONS

Conception and design of the study: ECH, NF and DvdV; Analysis of data: ECH, DvdV and DG; Drafting of the manuscript: ECH and DvdV; Drafting of the figures: ECH and DvdV; all authors critically revised and approved the final version of the manuscript.

## FUNDING INFORMATION

This research did not receive any specific grant from funding agencies in the public, commercial, or not‐for‐profit sectors.

## CONFLICT OF INTEREST STATEMENT

None of the authors has any conflict of interest to disclose. We confirm that we have read the Journal’s position on issues involved in ethical publication and affirm that thisreport is consistent with those guidelines.

## Supporting information


DataS1

FigureS1

FigureS2

TableS1

TableS2

## Data Availability

All relevant data are available from the corresponding author upon request. Raw imaging data are not publicly available due to data protection regulations.
