# Supplementary material for: Feasibility of high‐density electric source imaging in the presurgical workflow: Effect of number of spikes and automated spike detection
Source: Epilepsia Open. 2023 Jun 1;8(3):785–96. doi: 10.1002/epi4.12732 (PMC10472417; doi:10.1002/epi4.12732)
Supplement: Supplementary file 1 — DataS1 [file EPI4-8-785-s002.docx]

***Supplementary Methods***

***Visual versus semi-automated IED detection in hdEEG***

In preparation for the visual review, the hdEEG was filtered by a 1- to 70 Hz band-pass filter and a 50 Hz notch filter via EGI Net Station Tools.

The full-length, filtered hdEEG was then displayed by EGI Net Station Review. First, the IEDs were detected purely visually from the beginning to the end by one EEG experienced clinician (E.-C. Heide, aware of clinical information). All spikes (<70 ms) and sharp waves (70-120 ms) were marked on the IEDs’ peak using a conventional display (e.g., longitudinal bipolar or average montages of the 10–20 electrode array plus extended electrodes in the temporal chain, corresponding to the 2017 IFCN recommendations^21^). The time needed for the visual review of the filtered hdEEG was measured and rounded up in fifteen-minute steps. IEDs were visually classified into different groups based on their morphology and topography, at lobar resolution. If more than one IED population was found, the one with the highest amount of IEDs was selected for processing. Epochs containing the IED (+/- two seconds) were clipped and exported to Matlab for the further processing.

Additionally, the filtered, full-length hdEEG was processed by Persyst Spike Detector P14 (Persyst, San Diego, California, USA; non-clinical use, Version 14, Rev. C). We excluded IEDs with a pre-specified spike probability measure of less than 0.9, corresponding to the “low sensitivity” settings of the software^22^. The remaining IEDs were then grouped automatically into different IED clusters according to the electrode with the highest amplitude. Each cluster was then sorted by the IED amount and displayed as two-second event epochs. These epochs were reviewed by the same clinician (E.-C. Heide, aware of clinical information) at least six months after the visual scoring using an extended average reference montage of the 10–20 electrode as mentioned above. The automatically detected events that did not meet IED criteria such as artifacts and physiological EEG patterns were excluded. The operator time needed for reviewing all two-second event epochs was measured and rounded up according to a fifteen-minute interval.

In order to compare purely visual and semi-automated IED selection, sensitivity, positive predictive value and success rate of Persyst IED detection and Cohen´s $\kappa$ (kappa) were determined. If the peak of the (semi-)automated IED was within 50 ms before or after the manually marked peak of the visual IED they were predefined as equivalent IEDs. Sensitivity was defined by the amount of visual IEDs detected also by Persyst, whereas positive predictive value was the amount of IEDs among all detected events. Success rate was described as the probability to detect at least 30 IEDs among all events of one cluster. In addition, the Cohen´s $\kappa$ for inter-rater agreement analysis was used to observe the agreement reliability between Persyst´s IED automated detection and the IED selection made by the EEG-experienced clinician (E.-C. Heide) visually but also semi-automatically with Persyst. Therefore, one calculation was done for the visual versus semi-automated IED selection and other for the visual versus automated IED Persyst detection. It was calculated as

$$\kappa=\frac{\Pr\left( a \right)-\Pr(e)}{1- Pr(e)}$$

where Pr(a) is the relative observed agreement between selection conditions, and Pr(e) is the hypothetical probability of chance agreement^23-25^.

HdESI was performed with the purely visually detected IEDs of the cluster with the highest number of IEDs and comparable semi-automated IEDs. For this reason, the semi-automated IEDs clustered automatically by Persyst according to the channel with the highest amplitude were grouped visually by hemisphere and lobar resolution to allow comparison with visual hdESI. If more than one IED population was found, the one used for hdESI was visually determined. The IEDs of this group were clipped (+/- two seconds) and exported to Matlab for the further processing.

***MRI acquisition and preprocessing***

An individual presurgical high resolution T1 or MPRAGE 3-T MRI of the entire head was acquired in all subjects (Magnetom Prisma, Siemens Medical Systems). The acquisition of 3D structural T1 weighted images and FLAIR images followed these protocols: magnetization-prepared rapid acquisition gradient echo (repetition time = 1900 milliseconds, echo time = 2.26 milliseconds, field of view = 256 x 256 x 256 voxel, flip angle = 9°, voxel size = 1 x 1 x 1 mm) and FLAIR (repetition time = 5000 milliseconds, echo time = 280 milliseconds, inversion time = 1600 milliseconds, field of view = 256 x 256 x 256 voxel, flip angle = variable, voxel size = 0.5 x 0.5 x 1 mm). For postsurgical MRI a T1 image was also used. The presurgical T1 image was segmented and reconstructed using FreeSurfer (version 6.0.0, https://surfer.nmr.mgh.harvard.edu/).

***Determination of surgical reference***

Postsurgical T1 was coregistrated to the presurgical T1 by SPM12 using a “normalized mutual information” cost function. Since the resected volume is post surgically filled with cerebrospinal fluid, we determined the differences between the pre- and postoperative cerebrospinal fluid (CSF) tissue classes. This was done by binarizing (threshold > 0.1) both tissue masks and masking the postsurgical T1 CSF tissue class by the presurgical T1 CSF tissue class. This initial identification of the resection zone was again binarized (threshold > 0). In order to remove falsely identified differences of scattered voxels in the subarachnoid space we used a three-dimensional median filter (kernel: 3 x 3 x 3 mm). We applied a smoothing function with a 4 x 4 x 4 mm kernel to account for residual spatial differences at the edges of the resected volume. The resection zone mask was reviewed with reference to the pre- and postsurgical MRI and manually corrected if needed. Finally, a three-dimensional Gaussian filter with kernel of 9 x 9 x 9 mm was applied to the mask to remove sharp edges from manual editing.

The extent of the resection was determined clinically including non-invasive and, for some patients, invasive EEG diagnostics and included the surgical access routes. Given the fact that only patients with good post-operative outcome (Engel I) were included, it can be assumed that the resection zone contains the epileptogenic zone or, at least, the clinically relevant propagation areas.

***Source Reconstruction***

The selected IED epochs (both the purely visual as well as the semi-automated) were further processed and analyzed with Fieldtrip (https://www.fieldtriptoolbox.org/, version fieldtrip-20191127) running in Matlab (version 9.0, R2018b, Mathworks Inc.). An overview of the processing steps is displayed in Figure 1 (A-D).

The hdEEG epochs were 70 Hz low pass and 1 Hz high pass filtered. Also, a 50 Hz band pass filter was applied to account for line noise as well as its 100 Hz and 150 Hz harmonics. The sensor data was re-sampled to 250 Hz. Noisy and artifact epochs were visually rejected. Next, an independent component analysis was performed to identify and remove components with electrocardiogram and eye movement artifacts. The IED epochs were then visually checked and temporally aligned to the peak of the IED. All IED epochs were finally averaged over epochs of ± 2000 ms around the peak.

To create the individual head model, the FreeSurfer processed, intensity normalized MRI of each subject was used. A regular 5 mm volumetric grid was constructed in the CAT12 template (CAT12; Christian Gaser 2018, http://www.neuro.uni-jena.de/cat/) in MNI space. Those standardized volumetric grid points were transformed back to the individual anatomical space by using the inverted DARTEL transformation (DARTEL; SPM12; https://www.fil.ion.ucl.ac.uk/spm/software/spm12/). These volumetric grid points were further used for the source space for each subjects’ forward model. An individual boundary element model with three layers of different conductivity (scalp: 0.33S/m, skull: 0.004S/m, brain: 0.33S/m) was constructed using the ‘dipoli’ method implemented in Fieldtrip. For the sensor model, the sensors were spatially aligned to the anatomical T1 using anatomical landmarks of fiducial positions (left/right preauricular point, nasion).

The averaged IED time course was projected to each volumetric grid point using weighted minimum norm estimation (wMNE). This inverse method as well as the time point at 50 % rising phase were chosen for the source reconstruction according to literature.^26,27,7,8^ The 50 % rising-phase of the IED was defined algorithmically by the time point at which half of the amplitude was reached and visually approved for plausibility by an EEG-experienced clinician (E.-C. Heide). For each dataset, the regularization parameter λ (mean λ: 0.0051 ± 0.0051) was calculated based on the signal to noise ratio (SNR): λ = 1/SNR. The SNR was defined by the quotient of the amplitude at the spike peak of the IED and the standard deviation of the baseline of the averaged spike epoch. The electrophysiological amplitude distribution in all volumetric points was interpolated into a 3D MRI space with a brain mask in order to obtain a 1 mm x 1 mm x 1 mm volumetric space of source activity for the rising phase of the averaged epochs of the IED population with the highest amount of IEDs.

Source maxima were compared to sublobe(s) and to the resection itself. The grey matter was divided visually into 19 ‘sublobes’ per hemisphere based on the Lausanne parcellation atlas.^18,48^

In order to evaluate the effect of IED amounts on hdESI accuracy, the shortest distance between the resection zone and the voxel with the maximum amplitude of the source reconstruction was measured for different amounts of IEDs of each patient. Furthermore, the distance between the intraindividual hdESI maximum when all IEDs of an individual were included and the hdESI maximum of reduced amounts of IEDs was determined. In detail, source reconstructions of different amounts of IEDs were calculated iteratively with the use of increasing amounts of epochs for each patient. The number of epochs ranged from one to the individual maximum with an upper limit of 120 epochs. The intraindividual selection of IEDs when reducing the amount of IEDs was performed by 25 permutations per IED.

REFERENCE:

48. Daducci A , Gerhard S , Griffa A , Lemkaddem A , Cammoun L , Gigandet X , et al. The connectome mapper: an open-source processing pipeline to map connectomes with MRI . PLoS One . 2012 ; 7 : e48121
