# Supplementary figures and images for: Feasibility of high‐density electric source imaging in the presurgical workflow: Effect of number of spikes and automated spike detection
Source: Epilepsia Open. 2023 Jun 1;8(3):785–96. doi: 10.1002/epi4.12732 (PMC10472417; doi:10.1002/epi4.12732)

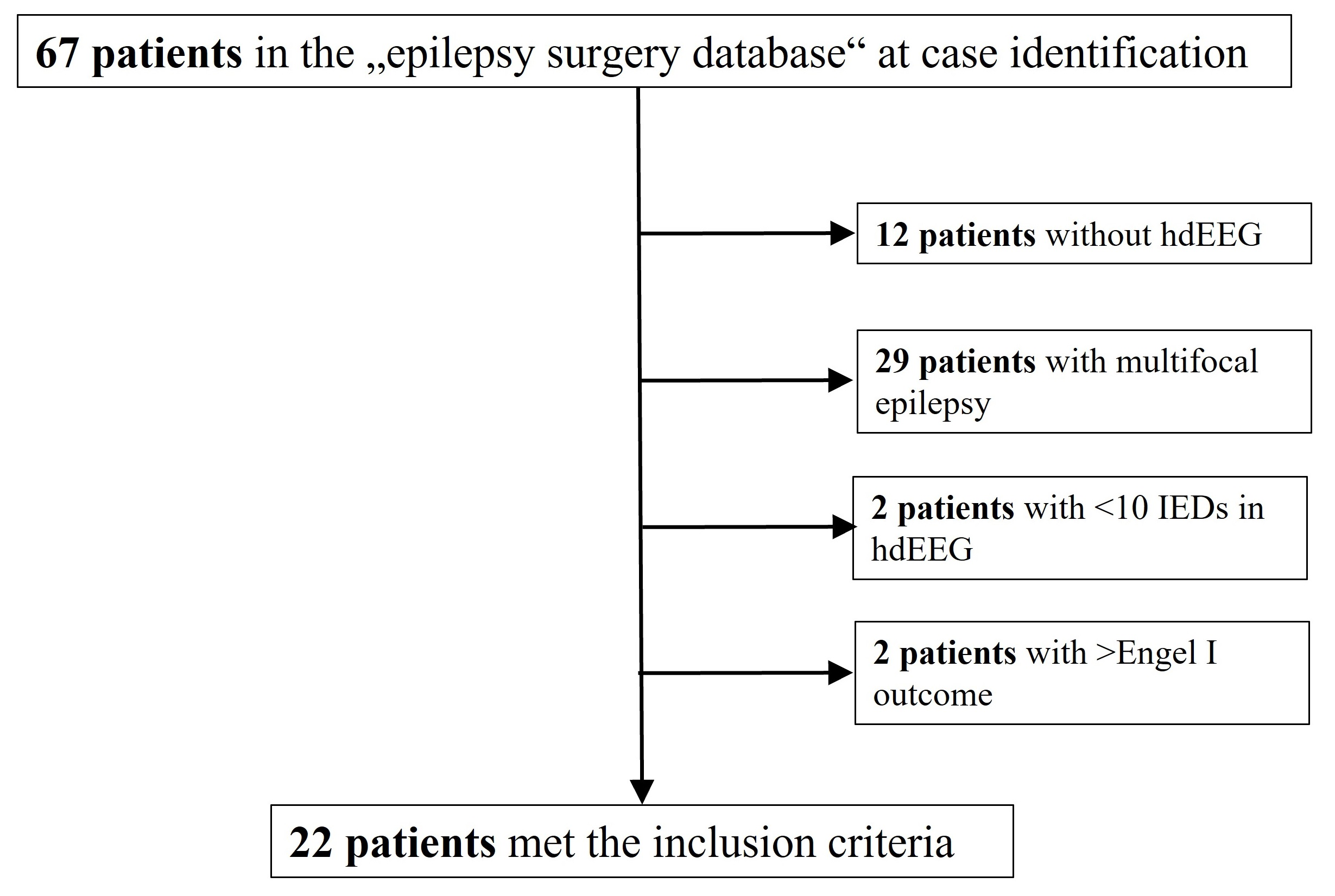

Supplement: Supplementary file 2 — FigureS1 [file EPI4-8-785-s005.tif]

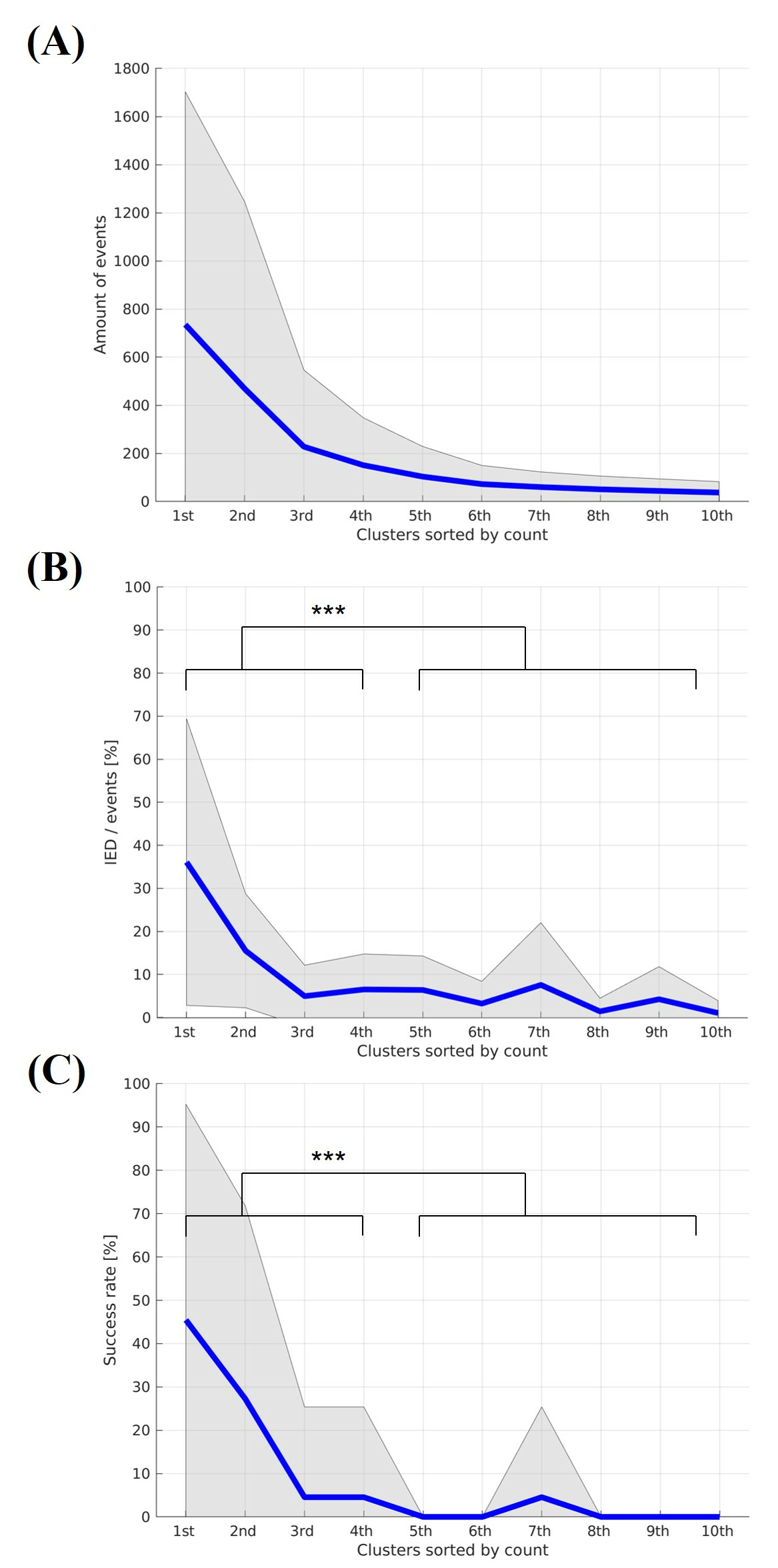

Supplement: Supplementary file 3 — FigureS2 [file EPI4-8-785-s001.tif]
